# Intimate Partner Violence (IPV) as a Public Health Crisis: A Discussion of Intersectionality and Its Role in Better Health Outcomes for Immigrant Women in the United States (US)

**DOI:** 10.7759/cureus.25257

**Published:** 2022-05-23

**Authors:** Sabina E Fridman, Nirmala Prakash

**Affiliations:** 1 Department of Integrated Medical Science, Florida Atlantic University Charles E. Schmidt College of Medicine, Boca Raton, USA

**Keywords:** intimate partner violence, public health, medical education, women's and adolescent health, immigrant health, domestic violence

## Abstract

Much of current work in providing care for intimate partner violence (IPV) in the United States (US) is centered around screening female patients. There is minimal work to tailor screening of IPV to marginalized patient populations such as immigrant women.

This discussion explores the need for non-stigmatizing, intersectional perspective in medicine, especially in working with diverse immigrant populations and in facing the public health crisis of IPV. We explore the needs in our healthcare education and practice for intersectionality.

By understanding the need for intersectionality, current best practices in IPV screening, and operationalizing of such perspectives and practices, we draw attention to healthcare needs for immigrant women and aim to increase understanding of IPV in medical education.

## Editorial

Healthcare systems in the United States (US) face significant challenges in addressing the health of vulnerable populations, such as immigrants and refugees, based on their respective risk factors. Particularly, the intersection of various health risk factors must be understood and applied to patient care in the context of intimate partner violence (IPV). IPV, defined by the World Health Organization as “behavior by an intimate partner or ex-partner that causes physical, sexual or psychological harm”, increases adverse health outcomes for affected individuals. Additionally, the World Health Organization recognizes that most common violence against women, IPV, is a public health concern globally [1]. Negative health outcomes are not isolated, but rather may be exacerbated by pre-existing health concerns and barriers to care. In the case of vulnerable immigrant women, health outcomes must be considered within the context of intersectionality: within the multiple social identities affecting patient health.

Major health organizations such as the Center for Disease Control and the US Preventative Services Task Force, with divisions aimed at preventing and screening for IPV, are still yet to adopt concrete, culturally appropriate screening recommendations for immigrants. The US must redesign its approach to understanding IPV in the context of immigrant women to prevent its own growing public health crisis. With appropriate consideration of the extent of patients’ experiences, barriers to good health, and the need for their trust in US health systems, healthcare teams must be better equipped to handle IPV in immigrant populations. 

As vulnerable patients, immigrant populations face detriment to the quality of their care due to socioeconomic background, immigration status, and policies limiting access to publicly funded healthcare. To provide appropriate, patient-centered care for immigrant women, it is imperative to consider the intersectionality of the patient’s experience as well as incorporate such considerations into medical education and training.

Academic medicine should adapt and longitudinally integrate bias recognition and dangers of stigmatization to marginalized patient populations in the curriculum of healthcare education. Training healthcare students to be mindful that their personal experiences and beliefs influence the care they provide is an effective, from-the-roots-up education that will result in more mindful, socially responsible clinicians. Educating future healthcare teams to address the effects of health issues within the context of an immigrant experience and addressing care with regards to the intersectionality of the patient at hand allows for more patient-centered care. 

IPV is multifactorial in nature. Immigrants to America, especially women, may bring with them a history of trauma, IPV, and other stressors from their home countries that impact their health. An analysis of the barriers to IPV screening for immigrant women in the US would be remiss without addressing the challenges faced by immigrant transgender women. Immigrant transgender women may face a barrier to care at the intersection of cumulative discrimination due to race, gender identity, and immigrant status. As they may come to the US with their own history of discrimination, they may be weary of trusting a new system. Immigrants such as transgender women of color, for instance, face tremendous barriers to care at the intersection of transgender discrimination, racism, and anti-immigrant bias. In addressing health concerns like IPV, it is important to recognize the cumulative exposures to discrimination and health burdens that pose further concern for marginalized patients.

For a healthcare team to appropriately mediate IPV and its associated health implications in immigrant women, it is vital to first identify signs and symptoms of exposure. Signs of exposure are not exceptionally different between immigrants and US-born patients. However, it is important to note additional factors that may negatively impact immigrant health. IPV risk specific to immigrant populations includes change in social status, longer time since emigration and continuation of social norms from country of origin. These risks may be further compounded by shared, marginalized identities that already face inadequate health screening and poor resource access. Thus, healthcare teams must be adaptive and vigilant in identifying all risk factors when considering IPV in female immigrant patients.

Intersectional perspective elicits viewing individuals at the intersection of their identities in order to understand the specific challenges they face [2]. In this regard, treating immigrant women as either purely immigrants, women, mothers, or any single identity, rather than the sum of all of their race, gender, and social identities can be dismissive of their complete experience. Unmet healthcare needs require attention to multiple, simultaneous risk factors to compensate for disparities in vulnerable patient populations. An intersectional perspective allows for more patient-centered, culturally competent, non-stigmatizing clinical experiences for vulnerable patients like immigrant women affected by IPV.

Certain good practice measures for diverse population care seem lacking from the inherent structure of medicine in America, and acknowledging these disparities is vital to bettering patient care. The next step must be to understand wherein gaps lie from medical education to training through to practice that result in practitioners that under-serve their patients. 

Healthcare teams should mitigate potential harms of screening for IPV which may expose shame, guilt, self-blame, partner violence, and repercussions of false-positive results. Innate bias of the clinician screening an immigrant patient with potentially no history of IPV, may allow for error, improper labeling, and further stigmatization of a patient. Additionally, a paternalistic or assumptive nature of screening may be insulting or intimidating to a patient that would otherwise benefit from support services. 

Discussing a topic as sensitive as IPV with any patient, much less a marginalized immigrant patient, requires proactive, appropriate communication strategies. Such communication abilities must be learned not in practice but through consistent emphasis in medical education. Addressing a patient using people-first language addresses them as more than their trauma, their experience, or their medical diagnosis and establishes a level of respect for the individual. Further communication strategies such as motivational interviewing, open-ended questions, and judgement-free responses are vital in providing patient-centered care. Researchers should also be intentional with language and aid to remove stigma from terminology made commonplace in the field through academic papers. 

Medical ethics is a valuable curriculum within medical education that helps future physicians develop skills to establish safe clinical environments when dealing with “gray areas” and uncomfortable topics in medicine such as end of life care, sexual health, and delivering bad news. IPV screening and discussion falls along the more uncomfortable topics to have with a patient, yet previous research has seen the gaps, locally and globally, in medical student competency and comfort in dealing with IPV when it comes to their patients [3,4]. Didactic lectures, both in pre-clinical and clerkship education, must include topics that emphasize immigration as a social determinant of health, women’s health concerns, and a clear understanding of intersectionality in the way it affects patients. Our future generation of healthcare practitioners must be comfortable in understanding a patient’s experience with gender, race, socioeconomic status, previous stigma and trauma to provide patients with thoughtful, humanistic care.

Although the US immigrant population has more than doubled in past decades, healthcare still faces considerable challenges in acknowledging the immigrant experience. Barriers to healthcare for immigrant women and the prevalence of IPV pose a public health crisis, to which the healthcare field must respond with thoughtful, tangible change. Implementing longitudinal training to normalize cultural sensitivity, intersectional perspective, language interpretation, and thoughtful follow-up may substantially impact the care that immigrant women, their children, and marginalized communities, of all backgrounds, receive. Taking care of the more vulnerable populations in the US and advancing the care of immigrant women affected by IPV will allow for advanced public health measures for the whole nation.
